# An Unusual Cause of Lower Gastrointestinal Bleeding: Cecal Dieulafoy's Lesion

**DOI:** 10.7759/cureus.7928

**Published:** 2020-05-02

**Authors:** Joseph Dailey, Michael B Russell, Mark Sterling

**Affiliations:** 1 Internal Medicine, Tufts Medical Center, Boston, USA; 2 Internal Medicine: Gastroenterology, Tufts Medical Center, Boston, USA

**Keywords:** colonoscopy, endoscopic treatment, lower gastrointestinal bleed, dieulafoy's lesion

## Abstract

Colonic Dieulafoy’s lesions are an exceptionally rare cause of lower gastrointestinal (GI) bleeding. These lesions are almost exclusively found in the upper GI tract based on previous reviews. We present a case of an 81-year-old man who presented with melena progressing to hematochezia and was found to have a cecal Dieulafoy’s lesion on colonoscopy. Hemostasis with clipping was achieved and allowed for the resumption of anticoagulation. This case demonstrates the importance of considering this diagnosis in lower GI bleeding when evidence of more common causes may not be present, especially considering these lesions amenability to endoscopic therapy.

## Introduction

Dieulafoy’s lesions are a rarely identified cause of gastrointestinal bleeding. They are defined as a dilated aberrant submucosal artery protruding through a small mucosal defect without primary mucosal ulceration or local aneurysm that does not undergo normal distal branching or tapering [1]. Studies estimate they are responsible for 1%-2% gastrointestinal (GI) bleeding. Based on reviews of case reports, only 2% of Dieulafoy’s lesions are located in the colon [2]. We present a case of a cecal Dieulafoy’s lesion successfully treated with hemostatic clipping. This case represents an extremely unusual etiology of a common presentation that is perhaps underdiagnosed and can be managed effectively with endoscopic hemostatic modalities.

## Case presentation

**Figure 1:**
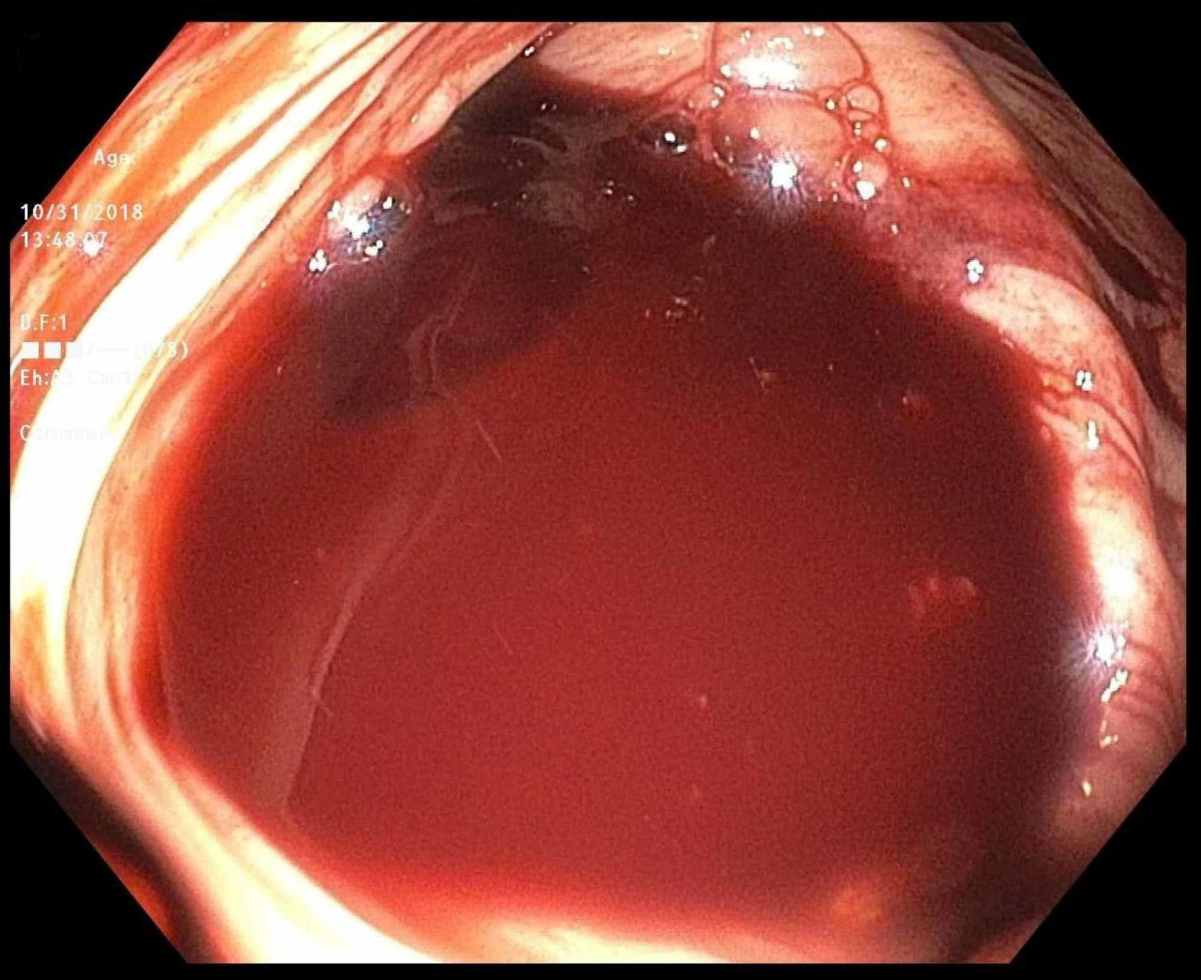
Colonoscopy Cecum with pooled blood.

**Figure 2:**
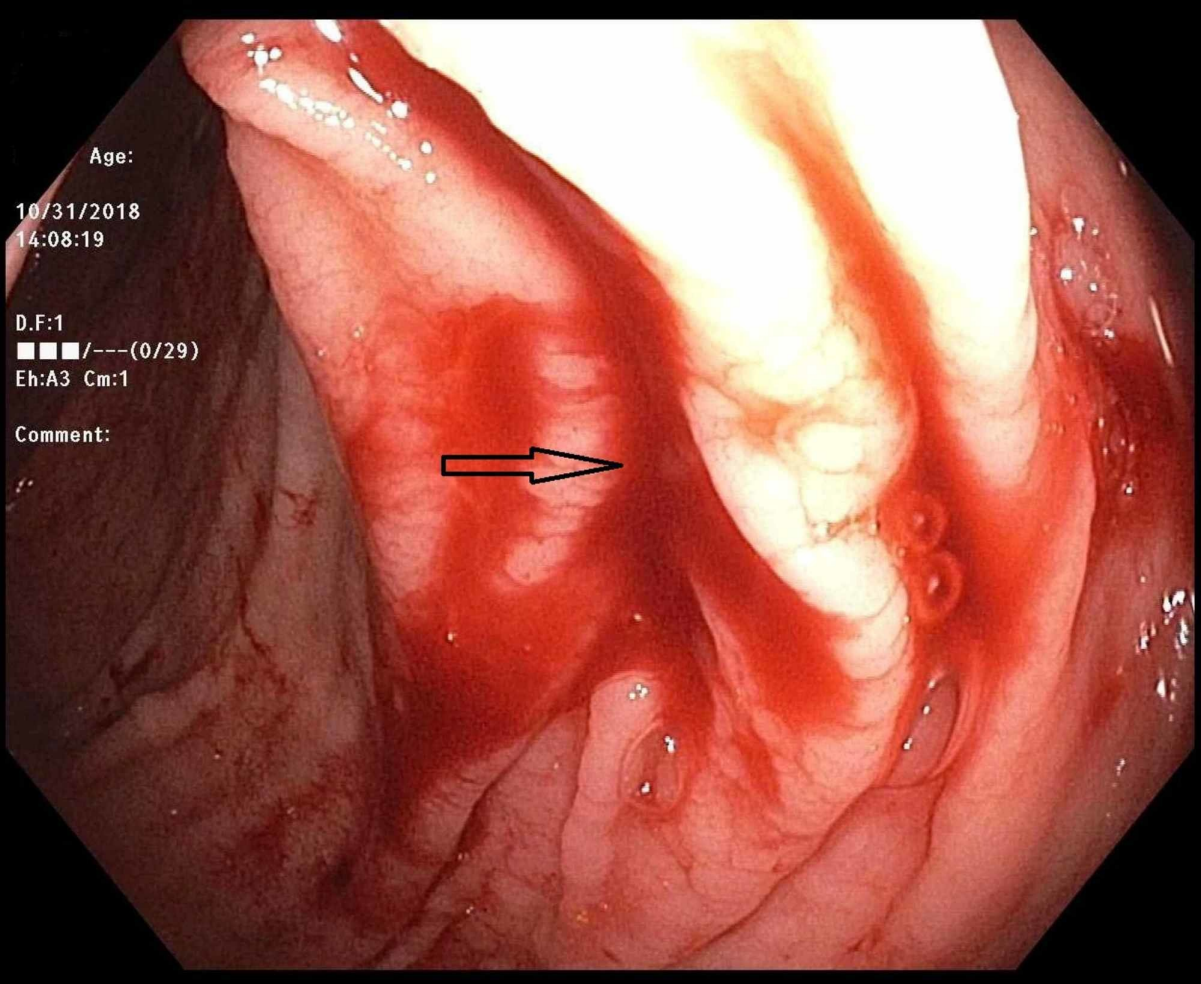
Colonoscopy Active bleeding source in the cecum.

**Figure 3:**
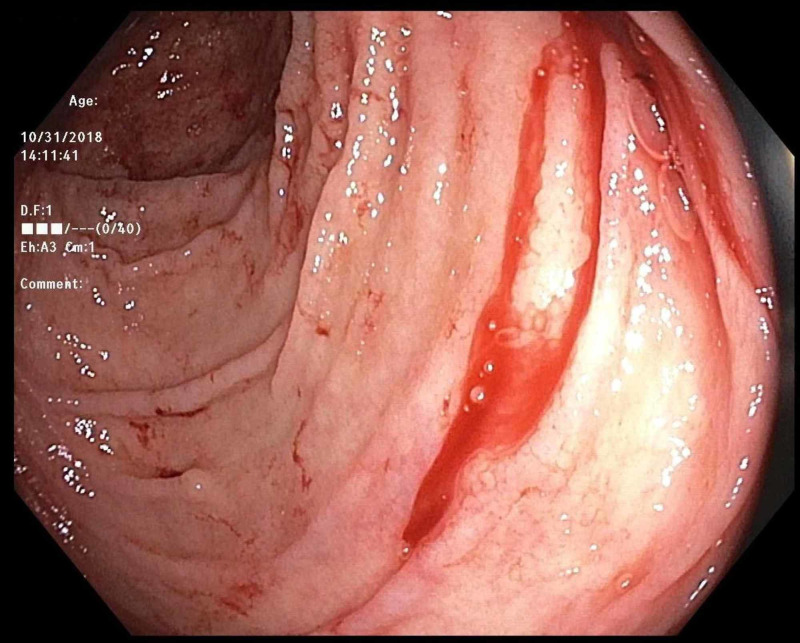
Colonoscopy post-washing Bleeding cecal Dieulafoy's lesion (top-right corner of the image).

**Figure 4:**
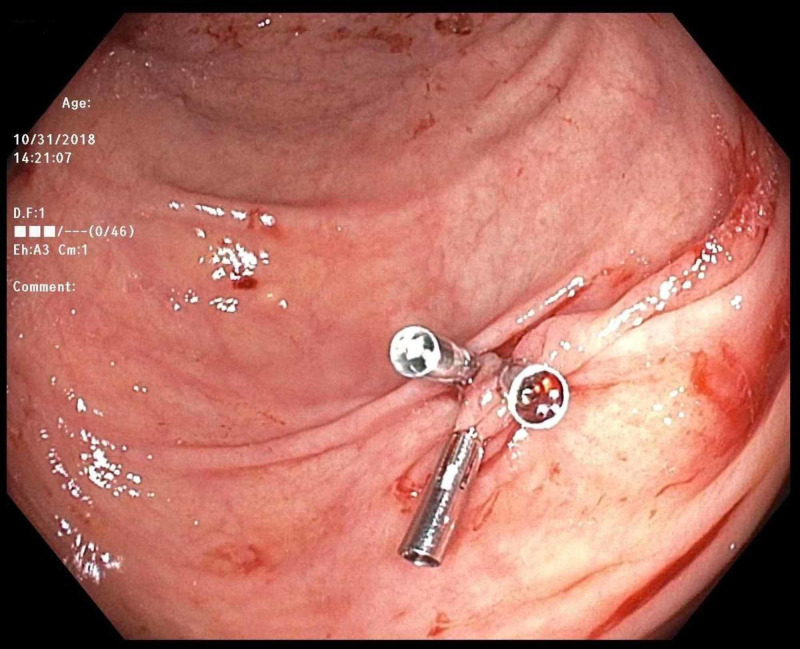
Colonoscopy Hemostasis achieved with three endoclips.

An 81-year-old man with a past medical history of hypertension and recently diagnosed pulmonary embolism presented with melena. His medications included ibuprofen for chronic back pain, as well as rivaroxaban, which he started one month prior to presentation. Since his hospitalization for pulmonary embolism, he was in his usual state of health until presenting with three days of melena without abdominal pain or hematemesis. He had never had bloody stools before, and his last colonoscopy three years prior had been unremarkable. Upon arrival, he was orthostatic with labs notable for hemoglobin 9.2 (down from baseline 12.5), international normalized ratio (INR) 1.5, platelets 99, blood urea nitrogen (BUN) 35, and creatinine 1.0. Outside hospital computed tomography (CT) abdomen/pelvis showed gastric wall thickening but an unremarkable liver and small/large intestine. CT pulmonary angiography (CTPA) showed a stable right lower lobe PE with a similar clot burden to prior imaging. Rivaroxaban was held, and on Day 2 of his hospitalization, he underwent upper endoscopy, which was normal. That day, he progressed to having maroon-colored hematochezia and his hemoglobin dropped to 7.2 for which he was transfused 1 unit of blood. On Day 3 of his hospitalization, he underwent colonoscopy, which showed bright red blood throughout the entire colon and two small right-sided polyps but no diverticulosis or evidence of colitis. After the aggressive washing of a large pool of blood in the cecum (Figure 1), an actively bleeding Dieulafoy’s lesion was seen adjacent to the ileocecal valve (Figure 2). There was no evidence of erythema, ulceration, or erosions at the site (Figure 3). Three endoclips were placed on the lesion with complete hemostasis (Figure 4). His hemoglobin remained stable, rivaroxaban was resumed the next day, and he had no further symptoms of GI bleeding through the two-month follow-up.

## Discussion

Our patient presented with melena progressing to maroon-colored hematochezia, which carries a wide differential. After ruling out a brisk upper GI bleed with upper endoscopy, we assessed for possible sources of lower GI bleeding, including diverticular bleed, hemorrhoids, colitis, and malignancy. Dieulafoy’s lesion was not highly considered before colonoscopy. Approximately 70% of these lesions arise in the stomach, with most in the lesser curvature under 6 cm from the gastroesophageal junction. Most of the remainder of the lesions come from the duodenum, with only 2% from the colon and 2% from the rectum [3]. Colonic Dieulafoy’s has only been described in a limited number of case reports, and there are no significant large case series describing them.

Colonic Dieulafoy’s lesions can present with melena, hematochezia, or bright red blood per rectum. In two case reports, there was a similar pattern to our patient. In one case, a 75-year-old female presented with two episodes of melena followed by one episode of bright red blood per rectum. She was found to have a transverse colon Dieulafoy’s lesion successfully treated with cautery and then two endoclips [4]. In another case from India, a 92-year-old male presented with multiple episodes of melena followed by one episode of blood per rectum. On colonoscopy, he was found to have a transverse colon Dieulafoy’s lesion treated again with cautery and two endoclips [5]. These presentations perhaps point out that right-sided colonic Dieulafoy’s lesions should be more highly considered in these types of mixed picture GI bleeds.

We found three case reports of cecal Dieulafoy’s lesions that have been reported. Two presented with painless hematochezia, and one presented with bright-red blood per rectum. Of these, two were effectively managed with clipping, while one was managed with cautery and epinephrine injection. The two cases managed with clips had no re-bleeding at six and 48 months, respectively; there is no data on the other case [6-8].

In the investigation of suspected lower GI bleeding, we feel these lesions are likely underdiagnosed given the challenge of finding them on colonoscopy. Visualization of the lesions can be impaired by the pooling of blood from aggressive bleeding or suboptimal bowel preps in the acute setting. Additionally, the likelihood of finding concomitant diverticula in the typical age range of patients with these bleeds is high, which perhaps leads to false attribution to diverticula as the etiology. In our case, it was only after a thorough investigation and washing of the colon that the cecal Dieulafoy's lesion was found.

The management of Dieulafoy’s lesions has evolved to where endoscopic measures can achieve hemostasis in up to 90% of cases [2]. Endoscopic therapy can be divided into three different modalities, namely, thermal, regional injection, and mechanical hemostasis [2]. Given the disease’s rarity, there are no large randomized trials comparing these techniques. In one small randomized trial of lesions in the upper GI tract, 12 patients were treated with mechanical means (nine clipping, three banding), and 12 with regional injection (epinephrine). Initial hemostasis was achieved in 91.7% of the mechanical group, and 75% of the injection group. None in the mechanical group went on to need surgery while 17% in the injection group did [9]. The consensus leans towards the mechanical hemostatic measure as the therapy of choice but other techniques appear acceptable.

## Conclusions

Our case describes an uncommon cause of GI bleeding occurring in an exceptionally rare location. In cases of lower GI bleeding when there is a lack of imaging or endoscopic evidence to suggest common etiologies, such as diverticula, colitis, hemorrhoids, or masses, colonic Dieulafoy’s lesions should be considered in the differential, especially considering the efficacy of endoscopic therapy in these lesions.

## References

[1] Lee Y, Walmsley RS, Leong RWL, Sung JJY (2003). Dieulafoy's lesion. Gastrointest Endosc.

[2] Baxter M, Aly EH (2010). Dieulafoy's lesion: current trends in diagnosis and management. Ann R Coll Surg Engl.

[3] Nojkov B, Cappel M (2015). Gastrointestinal bleeding from Dieulafoy’s lesion: clinical presentation, endoscopic findings, and endoscopic therapy. World J Gastrointest Endosc.

[4] Ma C, Hundal Hundal, R R, Cheng E (2018). Colonic Dieulafoy’s lesion: a rare cause of lower gastrointestinal hemorrhage and review of endoscopic management. Case Rep Gastrointest Med.

[5] Atri H (2018). Case report on colonic Dieulafoy’s lesion: a rare cause of lower gastrointestinal hemorrhage. International Conference on Digestive Diseases. Dubai, UAE. Dec 8.

[6] Saraireh H, Al Hanayneh M, Salameh H, Parupudi S (2017). Dieulafoy of cecum: a rare cause of refractory gastrointestinal bleeding in an uncommon location. Dig Liver Dis.

[7] Sone Y, Nakano S, Taked I, Kumada T, Kiriyama S, Hisanaga Y (2000). Massive hemorrhage from a Dieulafoy lesion in the cecum: successful endoscopic management. Gastrointest Endosc.

[8] Fukita Y (2013). Treatment of a colonic Dieulafoy lesion with endoscopic hemoclipping. BMJ Case Rep.

[9] Chung IK, Kim EJ, Lee MS (2000). Bleeding Dieulafoy's lesions and the choice of endoscopic method: comparing the hemostatic efficacy of mechanical and injection methods. Gastrointest Endosc.

